# Barriers Associated with Access to Prescription Medications in Patients Diagnosed with Type 2 Diabetes Mellitus Treated at Federally Qualified Health Centers

**DOI:** 10.3390/pharmacy10040079

**Published:** 2022-07-08

**Authors:** Asma M. Ali, Ewan K. Cobran, Henry N. Young

**Affiliations:** 1 Department of Family Medicine and Community Health, University of Wisconsin-Madison, 1100 Delaplaine Ct., Madison, WI 53715, USA; 2 Department of Clinical and Administrative Pharmacy, University of Georgia College of Pharmacy, Athens, GA 30602, USA; ecobran@uga.edu (E.K.C.); hnyoung@uga.edu (H.N.Y.)

**Keywords:** access to prescription medications, barriers, Federally Qualified Health Centers, type 2 diabetes mellitus

## Abstract

This study describes access to prescription medications and examines personal, financial, and structural barriers associated with access to prescription medications in patients with type 2 diabetes treated at Federally Qualified Health Centers. We used a cross-sectional design to analyze data retrieved from the 2014 Health Center Patient Survey. Adult participants who self-reported having type 2 diabetes were included in this study. Predictor variables were categorized into personal, financial, and structural barriers. Outcomes include being unable to get and delayed in getting prescription medications. Chi-square and multivariable regression models were conducted to examine associations between predictor and outcome variables. A total of 1097 participants with type 2 diabetes were included in analyses. Approximately 29% of participants were delayed, and 24% were unable to get medications. Multivariable regression results showed that personal barriers, such as federal poverty level, health status, and psychological distress were associated with being unable to get medications. Financial barriers including out-of-pocket medication cost and employment were associated with access to prescription medications. Type of health center funding program as a structural barrier was associated with access to medications. In conclusion, multi-level tailored strategies and policy changes are needed to address these barriers to improve access to prescription medications and health outcomes in underserved patient populations.

## 1. Introduction

Diabetes mellitus (DM) is a chronic disease of elevated blood glucose levels (hyperglycemia) [1,2]. More than 90% of patients with diabetes have type 2 DM [1]. Anti-diabetes medications constitute an important component of the collective management protocol needed to achieve glycemic control in patients with diabetes. Patients with type 2 DM are usually started on metformin, and other agents including insulin are added based on glycemic control and symptoms [3,4]. Some patients with type 2 DM are started on combination therapy for rapid attainment of glycemic goals [3]. Failure to effectively manage diabetes places patients at risk of developing microvascular and macrovascular complications as a result of hyperglycemia [5]. Continuous access to prescription medications is important for patients to achieve desired treatment outcomes and prevent adverse health outcomes (e.g., complications and hospitalizations) [6]. Encountering difficulties accessing prescription medications has been associated with high A1C levels [6,7].

The Institute of Medicine (IOM) defines access as the timely use of health services to achieve the best health outcomes [8]. The IOM proposes three types of barriers to access: (1) personal barriers that may prevent patients from seeking medical care including education, income, attitudes toward treatments, and psychosocial factors; (2) financial barriers that may limit access either by inability to pay for health services or by preventing healthcare providers from treating patients of limited resources (e.g., lack of insurance); and (3) structural barriers that are obstructions to medical care directly related to the organizational structure of healthcare providers, such as location of clinics [8]. Minority populations and patients from low socioeconomic status (SES) face more difficulties accessing health services compared to other patients [8]. Barriers such as lack of transportation, distance to care, inadequate health insurance, chronic conditions, and language can hinder access to needed healthcare services including prescription medications [9,10]. Moreover, psychological distress is associated with prescription refilling and diabetes health outcomes (e.g., A1C levels) [11,12,13]. Prevalence of serious psychological distress (SPD) (as measured by Kessler Psychological Distress Scale) in patients with diabetes is twice as much as patients without diabetes [14]. SPD is negatively associated with diabetes outcomes and the process of care [11,15,16]. Patients with diabetes and psychological distress are less likely to seek medical care and refill their prescription medications in comparison to patients with only diabetes [13]. Psychological distress has shown negative associations with glycemic control in patients with diabetes from underserved populations [11,17].

The Health Resources and Service Administration (HRSA) funds health centers to provide health services for indigent patients and individuals from underserved areas to improve access to healthcare resources and health outcomes. Federally Qualified Health Centers (FQHCs) are outpatient clinics that qualify for funding under Section 330 of the Public Health Service (PHS) Act from the HRSA Bureau of Primary Health Care (BPHC) [18]. FQHCs include Community Health Centers (CHCs), Migrant Health Centers (MHCs), Health Care for the Homeless (HCH), and Public Housing Primary Care (PHPC) [19]. These entities offer services to all people by establishing a sliding fee scale discount based on their ability to pay [18]. One in three people living in poverty rely on FQHCs for health services [20]. Approximately 91% of FQHC patients have a maximum income of 200% of the Federal Poverty Level (FPL) and 62% are from racial/ethnic minorities [21]. In 2020, approximately 2.6 million diabetes adults received health services at health centers around the US [21].

The BPHC requires FQHCs to offer specific services to improve patients’ access to care [19,22]. Some of the required health services are related to family medicine, internal medicine, pediatrics, obstetrics, or gynecology; diagnostic laboratory and radiologic services; and preventive health services [22]. Although there are no specific requirements for pharmacy services in FQHCs, some provide access to prescription medications [23,24]. Researchers found that publicly insured low-income patients treated at FQHCs were significantly less likely to be unable to access their medications because they could not afford them compared to patients treated at other health centers and private clinics [23]. FQHCs try to mitigate cost-related access barriers by offering medications at a significantly reduced price primarily through two programs: 340B drug pricing program and prescription assistance program (PAP) [25]. Yue et al., found that the use of enabling services (e.g., free medications, care coordination, transportation, and interpretation) by FQHC patients that address barriers to access, and social determinants of health was associated with improved use of primary care and preventative services and satisfaction with care [26]. However, patients treated at FQHCs who at least had one chronic condition faced barriers in accessing prescription medications more than those who had no chronic conditions [9]. Researchers reported that uninsured patients treated at FQHCs used a smaller number of prescription medications and were more likely to be unable to get their medications compared to patients who had Medicaid insurance [9,27]. These studies indicate that access to prescription medications may be an issue within FQHCs that warrants further investigation.

Additional research is needed to identify factors associated with access to prescription medications in patients with diabetes treated at FQHCs to inform strategies to help patients obtain and use medications to manage health conditions. The objectives of this study are to (1) describe access to prescription medications and (2) examine personal, financial, and structural barriers associated with access to prescription medications in patients with diabetes treated at FQHCs. This research uniquely identifies multiple barriers (including psychological distress) to prescription medications access within a national sample of patients diagnosed with type 2 diabetes and treated at FQHCs. This research will help uncover strategies and interventions at different levels of the healthcare system (e.g., clinical practice and policy) that could be implemented to overcome those barriers and improve health outcomes in patients with diabetes from underserved communities.

## 2. Materials and Methods

### 2.1. Study Design and Sampling

This is a retrospective study with a cross-sectional design using data from HRSA’s 2014 Health Center Patient Survey (HCPS) (latest data available) [28]. Data were collected nationally between October 2014 to April 2015 by face-to-face interviews with patients from four BPHC funded grant programs: CHC, MHC, HCH, and PHPC programs. The HCPS uses a three-stage nested structure sampling design. Sampling started by selecting health center grantees, then eligible health centers within each grantee, and finally eligible patients who had at least one visit and received one service during the last 12 months before the current visit. Participants from CHC programs accounted for 57% of those who completed the interviews. The HCPS provides patient-level data to help understand access to prescription medications in FQHCs. Patients were included in this study if they were 18 years or older and confirmed being diagnosed with diabetes by answering yes to the survey question “Have you ever been told by a doctor or health professional that you had diabetes or sugar diabetes?”.

### 2.2. Study Variables

Predictor Variables. Predictor variables were categorized into the three types of barriers proposed by the IOM [8]. Personal barriers include education (less than high school, high school, and more than high school), income as a percentage of the FPL (≤100%, 101–138%, 139–199%, 200–299%, and 300% or more), number of chronic conditions (response were categorized into 3 levels: 1 chronic condition, 2 chronic conditions, and 3 or more chronic conditions), use of insulin for diabetes treatment (yes/no), perceived health status which was examined by asking participants “Would you say your health in general is excellent, very good, good, fair, or poor?” (responses were categorized into two levels: poor/fair, good/very good/excellent), and psychological distress (responses were categorized into 3 levels: no/low psychological distress, mild/moderate psychological distress, and sever psychological distress). The 6-item Kessler Psychological Distress Scale (K6) was used to assess psychological distress in patients with diabetes. K6 is a valid with a high internal consistency reliability even when tested in a sample from an underserved population served by community health centers [29,30]. K6 assesses psychological distress by asking participants about 6 symptoms: “During the past 30 days, how often did you feel (1) so sad that nothing could cheer you? (2) nervous? (3) restless or fidgety? (4) hopeless? (5) that everything was an effort? (6) worthless?”. Each item was measured using 0–4 responses (0 = none of the time, 1 = a little of the time, 2 = some of the time, 3 = most of the time, and 4 = all of the time) [30]. Responses from the 6 items were summed to yield a score ranging from 0–24 with higher scores indicating higher psychological distress [30]. K6 scores were categorized using cut-off points suggested by Prochaska and colleagues into no/low psychological distress (K6 score = 0–4), mild/moderate psychological distress (K6 score = 5–12), and severe psychological distress (K6 score = 13–24) [31]. Financial barriers include employment status (yes/no), insurance status (yes/no), and out-of-pocket cost spent on prescription medications during the last year (0, USD 1–200, USD 201–600, USD 601 or more). Structural barriers include health center funding program (CHC, MHC, HCH, or PHPC), location medications filled (all of the medications filled at the health center or other), and area of the health center (urban or rural).

Covariates include age (18–44 years, 45–64 years, and 65 years and older), gender (male and female), and race/ethnicity (non-Hispanic White, non-Hispanic Black, Hispanic, and non-Hispanic other).

Outcome Variables. Outcome variables included two measures of participants’ access to prescription medications: (1) being unable to access medications, which was assessed by the question “In the last 12 months, were you unable to get prescription medicines you or a doctor believed necessary?”; and (2) being delayed in accessing medications, which was assessed by asking participants “In the last 12 months, were you delayed in getting prescription medicines you or a doctor believed necessary?”. Responses to access questions were dichotomous: no = 0 and yes = 1.

### 2.3. Statistical Analysis

Frequencies and weighted percentages (%) were calculated to describe all study variables. Respectively, Chi-square analyses and multiple regression models were conducted to examine bivariate and multivariable associations between predictor and outcome variables. Relative risk (RR) was calculated using modified Poisson regression rather than using logistic regression to calculate odds ratio (OR) because the strength of association can be overestimated with OR when the outcome is common [32]. To account for the complex design of the HCPS, strata, cluster, and weight variables provided in the survey data were included in the analysis. Significance level was set at a *p*-value of <0.05. *p*-values, and 95% confidence intervals (95% CI) were reported. SAS Enterprise Guide 7.1 was used to analyze the data.

## 3. Results

Of the total 1097 type 2 DM patients included in this study, 59% were White, 52% were female, and 51% were in age group 45–64. The majority of participants were not employed (73%), and had an income that was less than or equal to 100% of the FPL (59%). About 73% spent out-of-pocket money on prescription medications, 70% were insured, 64% perceived their health status as poor/fair, 47% had mild/moderate psychological distress, and 41% had no/low psychological distress. Approximately 29% of participants were delayed in getting prescription medications, and 24% were unable to get prescription medications. Full descriptions of study variables are presented in Table 1.

Chi-square analyses showed that personal barriers health status (*p* < 0.01), and psychological distress (*p* < 0.01) were significantly associated with being unable to get prescription medications. Using insulin for treating diabetes (*p* < 0.01), and health status (*p* < 0.01) were significantly associated with being delayed in getting prescription medications. Financial barrier out-of-pocket cost (*p* < 0.01) was significantly associated with being unable to get medications. Moreover, out-of-pocket cost (*p* < 0.01) and insurance (*p* < 0.01) were associated with being delayed in getting medications. Chi-square analyses results are presented in Table 1.

Multivariable regression results showed that participants who had an income equal to 200–299% of the FPL (RR: 0.42; 95% CI (0.24, 0.76)) were less likely to be unable to get their medications compared to those whose income was less than or equal to 100% of the FPL. Participants who perceived their health as good/very good/excellent (RR: 0.39; 95% CI (0.18, 0.84)) were less likely to be unable to get their medications compared to those who perceived their health as poor/fair. Participants who had mild/moderate psychological distress (RR: 2.01; 95% CI (1.28, 3.13)) were more likely to be unable to get their medications compared to those who had no/low psychological distress. Participants who were employed (RR: 1.97; 95% CI (1.21, 3.21)) were more likely to be unable to get their prescription medications compared to those who were not employed. Participants who spent out-of-pocket money on prescription medications were more likely to be unable to get prescription medications compared to those who spent zero dollars (USD 1–200 RR: 2.37; 95% CI (1.19, 4.69), USD 201–600 RR: 3.53; 95% CI (1.59, 7.85), and USD 601 or more RR: 5.16; 95% CI (2.62, 10.18)). Participants who received care from a Health Care for the Homeless program (RR: 2.19; 95% CI (1.03, 4.64)) were more likely to be unable to get their prescription medications compared to those who received care from a Community Health Center program

Participants who perceived their health as good/very good/excellent (RR: 0.54; 95% CI (0.30, 0.95)) were less likely to be delayed in getting their medications compared to those who perceived their health as poor/fair. Participants who spent out-of-pocket money on prescription medications were more likely to be delayed in getting prescription medications compared to those who spent zero dollars (USD 1–200 RR: 2.38; 95% CI (1.25, 4.55), USD 201–600 RR: 4.76; 95% CI (2.27, 9.99), and USD 601 or more RR: 3.16; 95% CI (1.57, 6.35)). Participants who received care from a Health Care for the Homeless program (RR: 2.31; 95% CI (1.27, 4.18)) or a Public Housing Primary Care program (RR: 2.32; 95% CI (1.25, 4.30)) were more likely to be delayed in getting their prescription medications compared to those who received care from a Community Health Center program. Table 2 presents the results of multivariable regression models.

## 4. Discussion

Although previous studies examined unmet needs in patients treated at FQHCs, no studies have focused on examining different barriers (including psychological distress) that might impact access to prescription medications, specifically in patients with diabetes. Identifying barriers to accessing prescription medications is important to help uncover strategies and interventions to overcome those barriers and improve health outcomes in patients with diabetes. This study assessed personal, financial, and structural barriers associated with access to prescription medications in patients with diabetes treated at FQHCs. Findings from this study suggest that multiple personal barriers are associated with diabetes patients’ access to prescription medications including FPL, health status, and psychological distress. Furthermore, out-of-pocket cost as a financial barrier and type of health center funding program as a structural barrier were associated with participants being unable and/or delayed in accessing their medications.

Results from this study demonstrated that participants who perceived their health as good/very good/excellent were less likely to be unable and delayed in accessing prescription medications compared to those who perceived their health as poor/fair. Previous studies that examined patients with chronic conditions who received care at FQHCs found similar results [9,27]. Findings from our study also suggest that mild/moderate psychological distress were associated with being unable to access prescription medications. Previous research found that patients with psychological distress faced barriers in accessing healthcare services in general [33,34]. A report by the CDC showed that New York adults with diabetes and psychological distress were more likely to face barriers filling prescriptions in comparison to those with only diabetes [13]. Furthermore, psychological distress was more likely in patients with diabetes than those without diabetes [13,14]. The ADA recommends continuous assessment of psychological distress in patients with diabetes and supports the provision of needed care to improve health outcomes [35]. Additional research is needed to gain a better understanding of patients with diabetes who are experiencing psychological distress in an effort to support self-management and overall well-being.

Study findings also showed that participants who had an income equal to 200–299% of the FPL were less likely to be unable to get their medications than participants with an income level of less than or equal to 100% of the FPL. Sliding fee scale discounts on required services as defined in Section 330(a)(1) of the Public Health Service Act are offered by FQHCs to provide a full discount for individuals and families with an income less than or equal to 100% of the FPL [36]. Of these services is the 340B medication discount program, however, not all FQHCs offer 340B program as a service [25]. Moreover, up until 2019, 12 states did not expand Medicaid eligibility under the Affordable Care Act (ACA), which leaves 2.4 million uninsured adults in the US with an income of 44–100% of the FPL [37]. These gaps in insurance and services provided by some FQHCs provide a plausible explanation for our findings. A recent study demonstrated that patients with diabetes in states that expanded Medicaid coverage reported significantly higher access to healthcare compared to states that did not expand coverage under the ACA [38]. Future efforts should examine strategies (e.g., policies to address fees and Medicaid coverage) aimed at improving this income group’s access to prescription medications.

Our findings demonstrated that employment status was associated with being unable to access prescription medications. It is possible that employed participants work in demanding jobs with strict schedules and limited benefits that restrict them from taking time off to get their medications [39]. Our results also suggest that participants who spent out-of-pocket money on prescription medications were more likely to be unable and delayed in getting prescription medications than those who did not spend any out-of-pocket money on medications. Costs associated with medications used to treat diabetes constitute 43% of the total direct economic burden of diabetes [40]. Although FQHCs try to mitigate cost-related access barriers by offering 340B drug pricing programs and PAP, they do not guarantee accessibility of these programs to all FQHC patients [25,41]. Shi et al. (2018) found that the number of 340B programs varies by state; in some states, the ratio of 340B programs to FQHC patients is low [25]. In addition, eligibility criteria for enrollment in PAPs exclude patients who are in need of this assistance (e.g., low-income and uninsured). PAPs also impose limitations on the medications that it covers (expensive specialty and brand names rather than less expensive generics) [42,43]. These factors might impose impediments on patients’ accessibility to medications. Efforts are needed to mitigate these barriers to minimize out-of-pocket spending on medications by patients treated at FQHCs.

Finally, our results showed that receiving care from a Health Care for the Homeless or a Public Housing Primary Care program was associated with delayed access to prescription medications. Liang et al., found that patients receiving care by HCH programs were more likely to face problems accessing prescription medications than patients receiving care by CHC programs [27]. Another study found that 36% of homeless patients receiving care by HCH programs experienced unmet needs for accessing prescription medications [44]. Factors associated with homeless patients’ unmet needs for accessing medications include age, out-of-home placement as a minor, employment during last year, lack of health insurance, and having two or more comorbid conditions [44]. Additional interventions are needed to improve access to prescription medications not only in CHC programs but also in other funded programs such as HCH programs.

## 5. Limitations

This study has some limitations that warrant mentioning. First, causation cannot be inferred between predictor variables and access to prescription medications due to the cross-sectional design of the study. Second, survey data were self-reported and collected by face-to-face interviews, which may be subject to recall and social desirability biases. Third, it is possible that there have been other potential factors (e.g., staff and availability of drug discount programs) that may be associated with access to medications that were not accounted for due to the use of already available secondary data. Finally, participants were included in the survey only if they had at least one visit during the last year; patients who needed care but were not able to access it were not included in the data (i.e., generalizability). However, HCPS’s sample represents patients who receive care at FQHCs.

## 6. Conclusions

In summary, this study highlights factors that may predict access to prescription medications in underserved patients with type 2 diabetes. Various factors including psychological distress and employment were associated with being unable to access prescription medications. Furthermore, perceived health status and out-of-pocket cost were associated with both being unable and being delayed in accessing medications. Multi-level tailored strategies and policy changes are needed to address these barriers to improve access to prescription medications and health outcomes in patients with diabetes treated at FQHCs.

## Figures and Tables

**Table 1:** Descriptive statistics and Chi-square analyses results for access to prescription medications (*n* = 1097).

Study Variables	Frequency	Weighted Percentage	Unable to Access *p*-Value	Delayed Access *p*-Value
***Age***			0.059	0.24
18–44 years	225	26.45		
45–64 years	685	51.02		
65 and older	187	22.52		
***Gender***			0.204	0.98
Male	415	48.01		
Female	682	51.99		
***Race***			**0.039**	**0.02**
Non-Hispanic White	265	59.44		
Non-Hispanic Black	241	16.88		
Hispanic	432	17.52		
Non-Hispanic Other	159	6.16		
**Personal and Cultural Barriers**				
***Education***			0.92	0.88
Less than high school	528	38.18		
High school	263	23.27		
More than high school	304	38.54		
***Federal Poverty Level***			0.19	0.14
Less than or equal to 100%	716	58.99		
101% to 138%	183	15.66		
139% to 199%	105	12.99		
200% to 299%	57	4.42		
300% or more	32	7.94		
***Insulin use to treat diabetes***			0.46	**0.001**
No	677	51.79		
Yes	420	48.21		
***Chronic conditions***			0.30	0.42
1 Chronic condition	209	17.34		
2 Chronic condition vs	475	38.32		
3 or more chronic conditions	413	44.35		
***Health status***			**0.002**	**<0.01**
Poor/Fair	703	64.19		
Good/Very Good/Excellent	394	35.81		
***Psychological distress (K6)***			**0.001**	0.32
No/Low psychological distress	473	40.63		
Mild/Moderate psychological distress	452	46.54		
Severe psychological distress	171	12.83		
**Financial Barriers**				
***Employment status***			0.22	0.28
No	817	72.79		
Yes	278	27.21		
***Insurance status***			0.09	**<0.01**
No	285	29.19		
Yes	810	70.80		
***Out-of-pocket money spent on medications***			**0.004**	**<0.01**
0	360	27.29		
USD 1–200	347	24.71		
USD 201–600	201	17.41		
USD 601 or more	166	30.58		
**Structural Barriers**				
***Health center funding program***			0.28	0.12
Community Health Center	682	94.03		
Health Care for the Homeless	181	2.80		
Migrant Health Center	146	2.44		
Public Housing Primary Care	88	0.73		
***Location medications filled***			0.97	0.39
All of the medications filled at the Health Center	273	17.43		
Other	788	82.57		
***Area of the health center***			0.29	0.14
Rural	365	61.31		
Urban	732	38.69		
**Outcome Variables**				
***Unable to get prescription medications***				
No	823	76.14		
Yes	226	23.86		
***Delayed in getting prescription medications***				
No	766	71.49		
Yes	282	28.51		

Note: Bold font numbers indicate statistical significance.

**Table 2:** Multivariable associations between barriers and access to prescription medications.

Predictor Variables	Unable to Access		Delayed Access	
	RR	95% CI	RR	95% CI
***Age***				
18–44 years	1		1	
45–64 years	1.42	0.84, 2.39	1.00	0.68, 1.48
65 and older	0.64	0.21, 2.01	0.6979	0.39, 1.62
***Gender***				
Male	1		1	
Female	1.07	0.68, 1.69	0.78	0.54, 1.14
***Race***				
Non-Hispanic White	1		1	
Hispanic	0.58	0.30, 1.14	0.88	0.46, 1.69
Non-Hispanic Black	0.87	0.45, 1.69	0.39	**0.18, 0.84**
Non-Hispanic Other	2.13	**1.15, 3.95**	1.51	0.82, 2.81
**Personal and Cultural Barriers**				
***Education***				
Less than high school	1		1	
High school	0.87	0.51, 1.48	1.20	0.67, 2.16
More than high school	1.25	0.70, 2.24	1.17	0.73, 1.88
***Federal Poverty Level***				
Less than or equal to 100%	1		1	
101% to 138%	1.42	0.88, 2.85	0.94	0.57, 1.54
139% to 199%	0.87	0.44, 1.68	1.47	0.89, 2.41
200% to 299%	0.42	**0.24, 0.76**	1.20	0.59, 2.43
300% or more	0.23	0.04, 1.35	0.28	0.049, 1.54
***Insulin used to treat diabetes***				
No	1		1	
Yes	0.85	0.56, 1.29	1.43	0.84, 2.44
***Chronic conditions***				
1 Chronic condition	1		1	
2 Chronic conditions	1.07	0.51, 2.27	0.93	0.21, 2.25
3 or more chronic conditions	0.67	0.29, 1.54	0.71	0.37, 1.38
***Health status***				
Poor/Fair	1		1	
Good/Very Good/Excellent	0.39	**0.18, 0.84**	0.54	**0.30, 0.95**
***Psychological distress (K6)***				
No/low psychological distress (0–4)	1		1	
Mild/Moderate psychological distress (5–12)	2.01	**1.28, 3.13**	1.67	0.99, 2.82
Severe psychological distress (13–24)	2.08	0.99, 4.37	1.22	0.65, 2.30
**Financial Barriers**				
***Employment status***				
No	1		1	
Yes	1.97	**1.21, 3.21**	0.89	0.54, 1.46
***Insurance status***				
No	1		1	
Yes	1.32	0.84, 2.11	0.87	0.58, 1.31
***Out-of-pocket money spent on medications***				
0	1		1	
USD 1–200	2.37	**1.19, 4.69**	2.38	**1.25, 4.55**
USD 201–600	3.53	**1.59, 7.85**	4.76	**2.27, 9.99**
USD 601 or more	5.16	**2.62, 10.18**	3.16	**1.57, 6.35**
**Structural Barriers**				
***Health center funding program***				
Community Health Center	1		1	
Health Care for the Homeless	2.19	**1.03, 4.64**	2.31	**1.27, 4.18**
Migrant Health Center	1.61	0.72, 3.58	1.34	0.718, 2.53
Public Housing Primary Care	1.92	0.98, 3.77	2.32	**1.25, 4.30**
***Location medications filled***				
Other	1		1	
All of the medications filled at the Health Center	1.01	0.57, 1.79	0.96	0.54, 1.69
***Area of the health center***				
Urban	1		1	
Rural	1.12	0.72, 1.74	1.22	0.74, 1.99

Abbreviations: CI, confidence interval. Note: Bold font numbers indicate statistical significance.

## Data Availability

The data used in this study are available on the Bureau of Primary Health Care website: https://bphc.hrsa.gov/data-reporting/health-center-patient-survey (accessed on 20 May 2022).
